# Memory of maternal temperatures: DNA methylation alterations across generations

**DOI:** 10.1093/plphys/kiae651

**Published:** 2024-12-18

**Authors:** Dechang Cao, Joke De Jaeger-Braet

**Affiliations:** Assistant Features Editor, Plant Physiology, American Society of Plant Biologists; Germplasm Bank of Wild species, State Key Laboratory of Plant Diversity and Specialty Crops & Yunnan Key Laboratory for Crop Wild Relatives Omics, Kunming Institute of Botany, Chinese Academy of Sciences, Kunming, Yunnan 650201, China; Assistant Features Editor, Plant Physiology, American Society of Plant Biologists; Department of Reproductive Epigenetics and Cell Biology, IST Austria (Institute of Science and Technology Austria), Klosterneuburg 3400, Austria

DNA methylation plays a crucial role in regulating gene expression and preserving genome stability. The dynamic DNA methylation levels throughout the life cycles of eukaryotes provide an orchestrated epigenetic regulation of cellular functions during development. 5-Methylcytosine (5mC) represents the most abundant DNA methylation in both plants and animals, which is sometimes regarded as the fifth base of eukaryotic genomes (Zhang et al. 2022). 5mC has been found to target both promoters and gene bodies at symmetrical CG and CHG sites and asymmetrical CHH sites, where H can be nucleotides A, C, or T (Mattei et al. 2022). The patterns of 5mC vary a lot across eukaryotes. Plants express considerable levels of CHG and CHH methylation, while vertebrate genomes are predominated by CG methylation, and 5mC may even be absent in some insects (Schmitz et al. 2019).

DNA methylation that occurs in plant embryos is established during embryogenesis, and maternal environments may exert substantial effects on the DNA methylation pattern (Mattei et al. 2022). A “memory” of maternal environments is crucial for the survival and adaptation of the offspring (Iwasaki et al. 2022; Sun et al. 2022). Considering the continuing changing climate, it is important to investigate whether temperature during embryogenesis might affect DNA methylation patterns and the subsequent post-embryo development of plants (Fig.). In this issue of *Plant Physiology*, Trontin et al. (2024) investigated temperature-induced memory during embryogenesis of maritime pine (*Pinus pinaster* Ait.) and found 10 potential candidate genes for the establishment of temperature memory.

**Figure.**
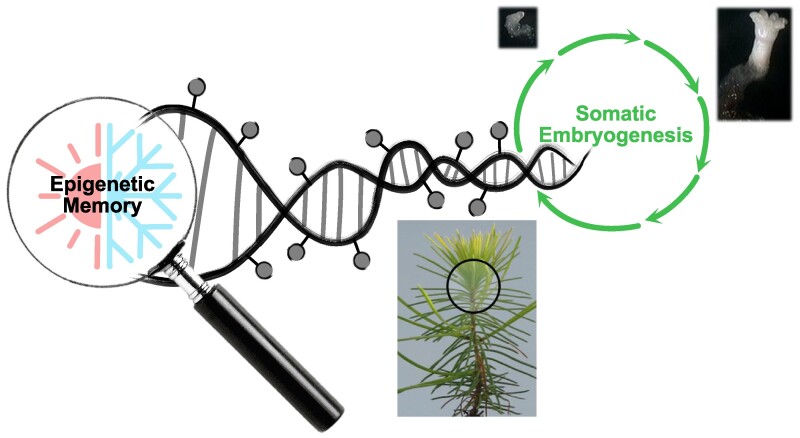
Illustration of the epigenetic memory of maternal temperatures. Representation of the investigation of DNA methylation alterations across generations using somatic embryogenesis of maritime pine (images from Trontin et al. (2024)).

Somatic embryogenesis provides an excellent research model to investigate the developmental transitions and DNA methylation dynamics during embryogenesis and post-embryonic growth. Trontin et al. (2024) incubated somatic embryos, initiated from the cryopreserved embryogenic line (PN519), at various temperatures (18 °C, 23 °C, 28 °C). The robust embryo culture method yields healthy mature embryos, which successfully develop into maritime pine seedlings. Compared with somatic embryos incubated at standard growth conditions (23 °C), embryos incubated at both 18 °C (considered as cold temperature) and 28 °C (considered as heat temperature) showed delayed maturation and altered sugar accumulation, suggesting considerable influence of the temperature on embryogenesis.

The authors analyzed global DNA methylation levels using high-performance liquid chromatography and performed gene model-specific methylome profiling using sequence capture bisulfite. During embryo development at standard growth conditions, early somatic embryos have less DNA methylation (15.1%) compared with further developed embryos, called cotyledonary somatic embryos (29.3%), whereas the DNA methylation level in the post-embryonic shoot apical meristem (SAM) exhibits an intermediate level (around 25%). Interestingly, the DNA methylation profile of early somatic embryo clustered closer with the profile of post-embryonic SAM rather than cotyledonary somatic embryos, suggesting alterations in DNA methylation modification during development. Despite the development-dependent changes of DNA methylation, no significant effect of temperature was detected on the overall DNA methylation levels. Thus, the different developmental phases undergo more pronounced DNA methylation modifications than the growth temperatures used in this study. In addition, clustering of the DNA methylation data aligned more with developmental stages than the temperature.

The authors further focused on the differential methylated cytosines (DMCs) in genes and promoters in response to growth temperature and developmental stage. The DMCs could be more frequently mapped to genes compared with promotor regions, particularly in response to temperature. Whether those DMCs lead to gene expression changed remains to be investigated.

The DMCs could be further grouped into 3 categories: DMCs that occur during either stage of embryonic development due to temperature (temperature-DMCs), maintained temperature-induced DMCs in SAM that were also detected in cotyledonary embryos (remaining temperature-DMCs), and DMCs due to developmental phase (development-DMCs). GO enrichment analyses showed that the DMC genes were highly associated with primary and secondary metabolism. Notably, unigenes containing the temperature-DMCs, remaining temperature-DMCs, and development-DMCs were most enriched in GO terms of photosynthesis, protein autophosphorylation, and polysaccharide metabolic processes, respectively.

Those memorized temperature-DMCs were more frequently detected in CG and CHG context compared with CHH, and the genes that maintain an epigenetic memory are related to stress responses and metabolic activities. In addition, the memorized temperature-DMCs were more prevalent under cold temperature compared with heat. In promotor regions, no temperature-dependent epigenetic memory seems to be observed in maritime pine, in contrast to a high retention rate of developmental memory in promoters, especially in the CG context.

Overall, the temperature effect on epigenetic memory was less pronounced than the developmental memory in this study of maritime pine. Considering that clustered DMCs are more likely to endow regulatory effects of a single gene, the authors screened genes with at least 5 temperature-induced DMCs and found several candidate genes for epigenetic control of temperature memory. The roles of those genes remain to be tested to further enhance our understanding of how plants retain memory of environmental conditions. In addition, with more diverse maternal environments and plant species investigated, in combination with the use of the recent advances in epigenetic technologies (Chen et al. 2023), the epigenetic secret of how plants adapt to the changing world might be unraveled soon.

## Data Availability

No new data were generated or analysed in support of this research.

## Dive Curated Terms

Replace("[{\"SHA_ENC_VALUE\":\"804365001ed81d96957260b7d33d65f01bd4f844\",\"IS_CURATED\":null,\"IS_SUBMITTED\":null,\"STANZA_VALUE\":null,\"ARTICLEID\":\"kiae651\"}]","","")
